# Novel motif associated with carbon catabolite repression in two major Gram-positive pathogen virulence regulatory proteins

**DOI:** 10.1128/spectrum.00485-24

**Published:** 2024-10-10

**Authors:** Jerry K. K. Woo, Adriana M. Zimnicka, Michael J. Federle, Nancy E. Freitag

**Affiliations:** 1 Department of Biopharmaceutical Sciences, Center for Biomolecular Sciences, College of Pharmacy, University of Illinois at Chicago, Chicago, Illinois, USA; The Hebrew University of Jerusalem, Rehovot, Israel

**Keywords:** CCR, *Streptococcus*, *Listeria monocytogenes*, virulence, PrfA, Mga

## Abstract

Carbon catabolite repression (CCR) is a widely conserved regulatory process that ensures enzymes and transporters of less-preferred carbohydrates are transcriptionally repressed in the presence of a preferred carbohydrate. This phenomenon can be regulated via a CcpA-dependent or CcpA-independent mechanism. The CcpA-independent mechanism typically requires a transcriptional regulator harboring a phosphotransferase regulatory domain (PRD) that interacts with phosphotransferase system (PTS) components. PRDs contain a conserved histidine residue that is phosphorylated by the PTS-associated HPr-His15~P protein. PRD-containing regulators often harbor additional domains that resemble PTS-associated EIIB protein domains with a conserved cysteine residue that can be phosphorylated by cognate PTS components. We noted that Mga, the PRD-containing central virulence regulator of *Streptococcus pyogenes*, has an EIIB^Gat^ domain containing a cysteine that, based on the presence of a similar motif in glycerol kinase, could be a target for phosphorylation. Using site-directed mutagenesis, we constructed phospho-ablative and phospho-mimetic substitutions of this cysteine and found that these substitutions modify the CCR of the Rgg2/3 quorum-sensing system. Moreover, we provide genetic evidence that the phospho-donor of this cysteine residue is likely to be ManL, the EIIA/B subunit of the mannose PTS system. Interestingly, a structurally distinct virulence gene regulator, PrfA of *Listeria monocytogenes*, harbors a similar cysteine-containing motif, and phospho-ablative and phospho-mimetic substitutions of the cysteine-altered CCR of PrfA-dependent virulence gene expression. Collectively, our data suggest that phosphorylation of a cysteine within the shared novel motif in Mga and PrfA may be a heretofore missing link between cellular metabolism and virulence.

**IMPORTANCE:**

In this study, we identified a novel cysteine-containing motif within the amino acid sequence of two structurally distinct transcriptional regulators of virulence in two Gram-positive pathogens that appears to link carbon metabolism with virulence gene expression. The results also highlight the potential post-translational modification of cysteine in bacterial species, a rare and understudied modification.

## INTRODUCTION

Bacterial cells are known for their metabolic versatility, being able to adapt and use a variety of sugars as carbon sources to facilitate growth. Carbon catabolite repression (CCR) is a mechanism that represses the use of less preferred sugars when a more desirable one is present (1). In firmicutes, CCR is exerted via two mechanisms, one being CcpA dependent and the other CcpA independent (Fig. S1) [reviewed in reference (1)]. For the CcpA-dependent mechanism, a conserved transcriptional repressor CcpA forms a complex with phospho-carrier protein HPr that is phosphorylated on a serine residue (HPr-Ser46-P), and this complex represses the expression of genes involved in the import and subsequent usage of alternative carbon sources when the preferred carbohydrate glucose is present in abundance. The CcpA-independent mechanism of CCR also involves HPr, but in this case, HPr is phosphorylated on a histidine residue (HPr-His15~P) rather than serine. HPr-His15~P primarily regulates the import of carbohydrates through a phosphotransferase system (PTS), such that phosphate is sequentially transferred from HPr-His15~P to EIIA and then to EIIB subunits that ultimately phosphorylate the incoming carbohydrate imported by cognate EIIC/D membrane transporters. In addition to CCR, HPr-His15~P also fulfills a regulatory function by phosphorylating transcriptional regulators that contain recognition domains known as PTS regulatory domains or PRDs (1). These transcriptional regulators are often dedicated to the regulation of genes encoding metabolic enzymes as well as the PTS components involved in the transport and metabolism of distinct carbohydrates.

The archetypical example of a PRD-containing transcription regulator is MtlR, which is conserved in several firmicutes. MtlR regulates the enzymes responsible for the metabolism of mannitol as well as the PTS mannitol transport components (PTS^Mtl^) (2–5). MtlR contains a DNA-binding domain at its N-terminus followed by two PRD domains, another domain that resembles an EIIB component of the galactitol family (EIIB^Gat^), and ends with a domain that resembles an EIIA component (4). The EIIB^Gat^ and EIIA domains of MtlR do not participate in the import of mannitol but rather their structural fold resembles that of the EII components of the galactitol family. Each of these domains, with the exception of EIIA in MtlR, is subject to phosphorylation: (i) HPr-His15~P phosphorylates both PRDs; (ii) EIIA subunits of PTS^Mtl^ phosphorylate the MtlR EIIB^Gat^; and (iii) the combinatorial phosphorylation of all of these domains serves to modulate the activity of MtlR in response to the presence of mannitol in either the presence or absence of glucose (5). The intricacy of this regulatory system with its multiple phosphorylation cascades highlights the complexity of CCR as bacterial cells tailor their gene expression to overcome nutritional stress.

Although PRD-containing transcriptional regulators often regulate the expression of cognate PTS components and substrate-specific enzymes for catabolism, some pathogens make use of these regulators to control the expression of virulence genes, recently referred to as PRD containing virulence regulators (PCVRs) (6). PCVRs serve to link virulence gene expression to cellular metabolism and nutrient availability. PCVRs, such as AtxA of *Bacillus anthracis*, MafR of *Enterococcus faecalis*, Mga*spn* of *Streptococcus pneumoniae,* and Mga of *Streptococcus pyogenes* (also known as Group A *Streptococcus* or GAS) contain similar domain structures as described above for MtlR [reviewed in reference (6)]. It appears thus far that PCVRs only interact with the centrally shared HPr protein, specifically HPr-His15~P, to regulate the transcription of virulence factors in response to carbohydrate availability via phosphorylation of the conserved histidine residue within the PRDs (7). It remains to be determined, however, if any carbohydrate-specific PTS components interact with PCVRs, owing to the presence of EIIB^Gat^ domains as observed for MtlR and its cognate PTS^Mtl^ (2, 3, 5).

Bacterial pathogens that coordinate virulence gene expression with carbon source availability include the human pathogen GAS, which typically colonizes the upper respiratory tract and skin surfaces. Colonization of these niches is assisted by the Rgg2/3 quorum-sensing (QS) system (8, 9) that aids in immune suppression, lysozyme resistance, and biofilm formation (10–12). The Rgg2/3 QS system has been extensively characterized; *rgg2* encodes a transcriptional activator, while *rgg3* encodes a transcriptional repressor of this QS system (12). During routine laboratory growth, the QS system remains off due to Rgg3-mediated repression of both *shp2* and *shp3*, encoding the pheromones of the system (12, 13). When synthetic SHP peptides are added into the medium, they are transported into the cell and bind to both Rgg2 and Rgg3, resulting in a change in Rgg-mediated regulation. SHP-bound Rgg3 has reduced DNA-binding affinity, releasing it from the promoter region of both *shp2* and *shp3*. This allows Rgg2-SHP complexes to access *shp* promoters and stimulate transcription, resulting in a positive feedback loop for this system (12).

**Fig 1:**
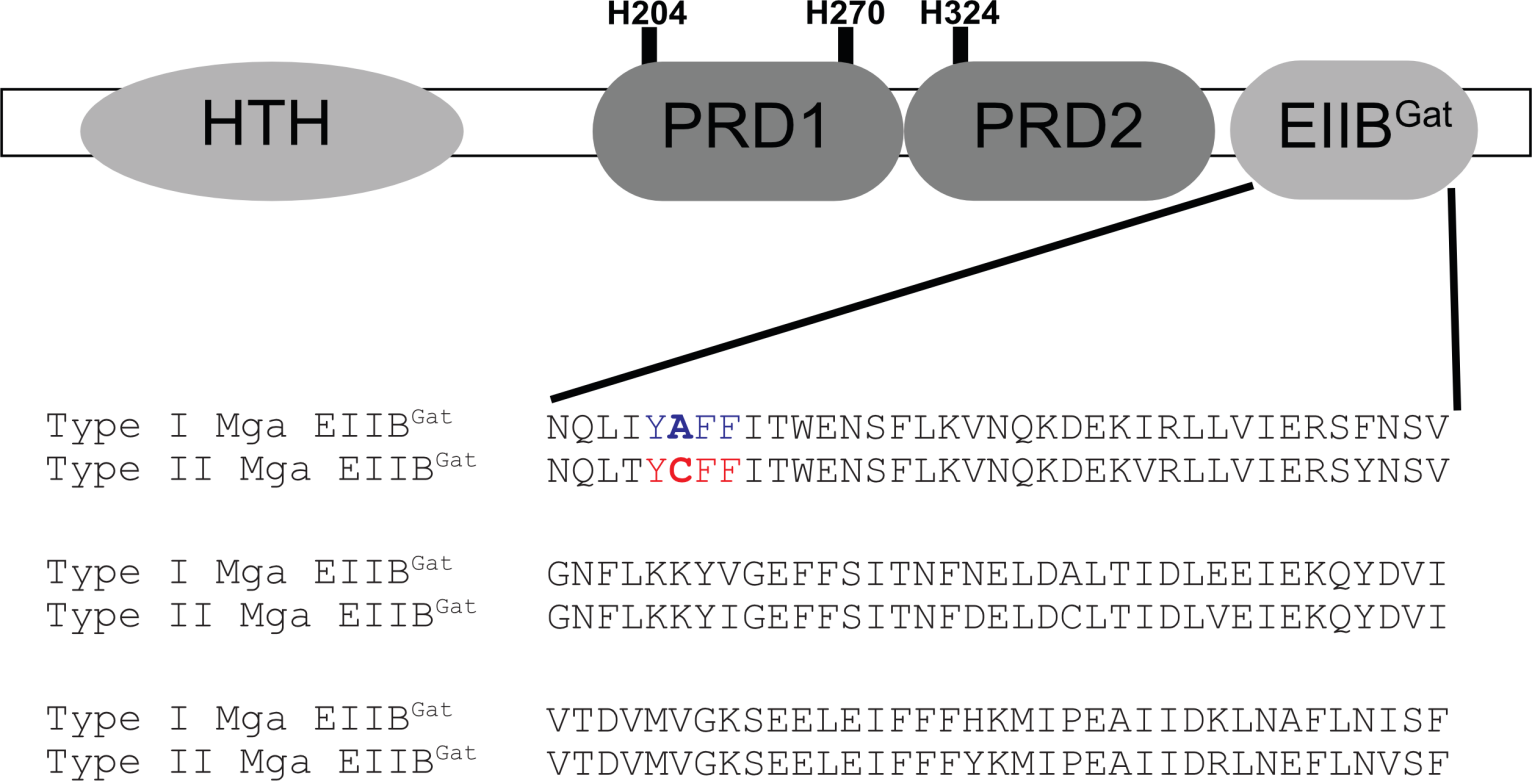
The structural domains of Mga and amino acid sequence alignment of EIIB^Gat^ domain between Type I and Type II lineages. The structural domains of both Type-I and Type-II Mga are highly conserved, but the amino acid alignment of the EIIB^Gat^ domain between them (383–493 a.a) revealed the presence of a cysteine residue in the latter lineage. The residue is gated by a triad of aromatic amino acids.

We previously characterized the environmental signals that influence the GAS Rgg2/3 QS system and found that it is highly induced when mannose is the sole carbon source, and its induction is suppressed in the presence of non-mannose carbohydrates such as sucrose or glucose, an overall hallmark of CCR (14, 15). The CCR exerted on the Rgg2/3 QS system is facilitated through a CcpA-independent mechanism that is reliant on ManL, the EIIA/B subunit of the PTS^Man^ (15). Interestingly, CcpA-independent CCR of the Rgg2/3 QS also requires Mga, the virulence gene regulator of GAS [(15) and Fig. S2] (Fig. 1). The domain architecture of Mga is highly similar to that of MtlR, such that the N-terminal portion of Mga harbors the DNA-binding domain, followed by two PRDs that are phosphorylated by HPr-His15~P on conserved histidine residues to modulate Mga activity, thereby tailoring the expression of virulence genes regulated by Mga in response to nutritional stress (7). Finally, like MtlR, Mga contains an EIIB^Gat^ domain at its C-terminus (Fig. 1), which in MtlR is phosphorylated on a conserved cysteine residue by its cognate EIIA component (3, 5). Phosphorylation of the MtlR cysteine within its EIIB^Gat^ domain provides a regulatory checkpoint for MtlR activation and indicates that the available carbohydrate is not a substrate for PTS^Mtl^, thus ensuring that MtlR remains inactive (5). It is possible that phosphorylation of the cysteine in the Mga EIIB^Gat^ domain could similarly serve as a regulatory checkpoint for the suppression of Rgg2/3 QS when mannose is not available. It should be noted that two different alleles of Mga exist within the GAS population: Type I and Type II Mga (16). The Type I Mga allele lacks a cysteine, while the Type II allele contains a cysteine residue within its EIIB^Gat^ domain. It remains to be determined whether the cysteine in Type II Mga can be phosphorylated. Moreover, the molecular mechanism that links both Mga and ManL with CCR on Rgg2/3 QS in GAS remains to be elucidated.

A second bacterial pathogen that uses CcpA-independent CCR to regulate the expression of virulence factors is *Listeria monocytogenes* (*Lm*) (17). The expression of *Lm* virulence factors depends on the master regulator of virulence, PrfA [reviewed in reference (18)], which belongs to the Crp/Fnr family of transcriptional regulators (19). The archetype of this protein family is Crp, which has been shown to mediate CCR in Gram-negative enteric bacteria, such as *Escherichia coli* and *Salmonella typhimurium* (20). To date, numerous efforts have been made to decipher the underlying molecular mechanism for CCR on PrfA activity. The constitutively expressed mannose PTS system known as PTS^mpo^, the inducible mannose PTS system known as PTS^mpt^, and ManR, the intermediary transcriptional regulator between these two PTS systems (21), have been suggested to influence PrfA activity. It has also been proposed that the metabolic state of the cell, as opposed to the presence of less-preferred carbohydrates, regulates PrfA activity (22). When *Lm* cells are grown in the presence of a preferred carbohydrate such as glucose or mannose, the majority of HPr exists as HPr-Ser46-P, which is reported to suppress the activity of PrfA (23, 24). However, when cells are grown solely in the presence of a less-preferred sugar, this leads to a decreased abundance of HPr-Ser46-P and an increased abundance of HPr-His15~P (25). HPr-His15~P consequently phosphorylates all available PTS permeases, priming the cells for the uptake of less-preferred sugars that are available (26, 27) and also appears to increase PrfA activity (22). Collectively, these studies highlight the mechanistic complexity that *Lm* adopts to ensure proper expression of PrfA-dependent virulence factors using the available carbohydrate as an environmental signal. Although a unified model that integrates all these observations has been proposed (28), it remains to be determined how PrfA actually perceives changes in nutritional status and carbohydrate availability to regulate its activity.

Here, we propose a novel mechanism by which GAS Mga and *Lm* PrfA may be linked to carbon availability. A critical cysteine residue may function in both regulators as a phospho-acceptor site to regulate both Mga- and PrfA-dependent gene expression in response to specific sugars. This cysteine-containing motif also appears to be present in select transcriptional regulators homologous to Mga in other bacterial species as well as in PrfA, which is structurally distinct from Mga. We propose that this motif may be the missing link that permits Mga and PrfA to detect metabolic shifts and carbohydrate availability and consequently regulate the activity of these two central virulence regulators in a carbohydrate-dependent manner.

## MATERIALS AND METHODS

### Bacterial strains and growth condition

The complete list of strains and plasmids used in this study is listed in Tables S2 and S3, respectively. *Streptococcus pyogenes* NZ131 (GAS) (M49 Serotype) was routinely grown in Todd Hewitt Broth (BD) supplemented with 0.2% yeast extract (Amresco) (THY) at 37°C. *Listeria monocytogenes* 10403S was routinely grown in Brain-Heart Infusion (BHI) (BD). When appropriate, antibiotics were added at the following concentration: erythromycin (Erm; 0.5 µg mL^−1^ for GAS) or chloramphenicol (CM; 7.5 µg mL^−1^ for *Lm*). Chemically defined medium (CDM) was prepared as previously described (12) and glucose (CDM-Glu) was added to a final concentration of 1%, vol/vol. For mannose (CDM-Man) or sucrose (CDM-Suc), either carbohydrate was added to a final concentration of 1%, vol/vol, in addition to glucose at a final concentration of 0.02%, vol/vol, unless otherwise stated, to support robust growth. *Escherichia coli* cloning strain BH10C (29) was routinely maintained in Luria broth (LB) or on Luria agar and if necessary, supplemented with erythromycin (500 µg mL^−1^).

### Construction of Mga point mutation complementation constructs

All constructs (pJW146–pJW147) were made using inverse PCR and plasmid pJW107 (15) as a template and with targeted mutations for the coding sequence of the cysteine residue within the primer listed in Table S4. Following PCR amplification, the constructs were ligated and sequenced to ensure the correct mutations were obtained. The constructs were transformed into electroporation-competent GAS Δ*mga* mutants or Δ*mga* Δ*manL* mutants for integration and passaged in THY at 30°C as previously described for the restoration with these *mga* variants (15). Verification of plasmid integration within the genome of GAS at the *mga* locus was performed with PCR using primer pairs of JWP0029 and JWP0008 to verify integration at the upstream region, and JWP0030 and JWP0007 for the downstream region. Positive integrants were stored as glycerol stocks at −80°C. A single colony of selected integrants was cultured in THY broth overnight and subsequently serially passaged in THY broth for 3 days at 30°C. The passaged culture was serially diluted and plated onto THY plates. Putative isolates were patched onto both THY-Erm plates and THY, and isolates that were Erm sensitive were verified for the restoration of *mga* allele and confirmed with PCR using primers JWP0029 and JWP0030. All positive variants were stored as glycerol stocks at −80°C.

### Generation of prfA point mutation complementation constructs

All constructs (pJW159 and pJW160) were made using inverse PCR and plasmid pNF1019 (30) as a template; pNF1019 is a pPL2 site-specific phage integration plasmid containing a wild-type copy of *prfA* with all promoters required for expression (30). Targeted mutation of the coding sequence for the cysteine residue was performed using primers listed in Table S4 and inverse PCR. The resulting plasmids isolated from *E. coli* were sequenced to verify the accuracy of the targeted mutation. The constructs were conjugated into a Δ*prfA* mutant (NF#1003) and selected for growth on Listeria Brilliance Agar (Oxoid) supplemented with 7.5 µg mL^−1^ chloramphenicol. Positive clones were verified for genomic integration of the construct using primers PL95 and NC16 as described (31).

### Luciferase assay

Strains of interest were grown overnight in THY broth at 37°C. The next morning, overnight cultures were diluted 1:10 in fresh THY broth and incubated at 37°C until the mid-exponential phase (OD_600_ = 0.2–0.4). Two milliliters of the cultures were washed twice with phosphate-buffered saline (PBS), resuspended in 200 µL of PBS, and inoculated into fresh CDM at a starting OD_600_ of 0.05. Cultures were incubated at 37°C without shaking, and the OD_600_ was monitored hourly using Genesys30 (Thermo Scientific). At the corresponding time point, 50 µL of culture was transferred to a 96-well white opaque plate (Greiner Bio-one), exposed to decyl aldehyde fumes for 30 s, and counts per second (CPS) were quantified using a Veritas microplate luminometer (Turner Biosystems). Relative light units (RLUs) were calculated by normalizing CPS to OD_600_. Each experiment was performed at least in triplicate on independent days.

### Quantification of LLO-associated hemolytic activity

Overnight cultures of *Lm* and its respective variants and mutants were diluted 10-fold in a fresh medium of choice and incubated at 37°C with shaking for 5 h. The optical density of the culture was measured and normalized to an OD_600_ of 0.5. Subsequently, 1 mL of normalized cells was centrifuged, and the supernatant was collected to assay for listeriolysin O (LLO)-associated hemolytic activity using phosphate-buffered saline-washed sheep erythrocytes (Cocalico Biologicals Inc., Reamstown, PA, USA) as previously described (32). Hemolytic activity was determined as the reciprocal of the corresponding serial dilution of culture supernatant, where 50% lysis of the erythrocytes was observed.

### Measurement of β-glucuronidase activity

Overnight cultures of *Lm* and its respective variants and mutants were diluted 10-fold in a fresh medium of choice and incubated at 37°C with shaking for 5 h, and the optical density of the culture was recorded at 600 nm (OD_600_). Subsequently, 5 mL of culture was centrifuged, and the cell pellet was resuspended in 500 µL of resuspension buffer (50 mM phosphate buffer; pH 7.0, 1 mM EDTA). The cell suspension was transferred to a screw-capped tube containing zirconia beads (0.2 µm) and subjected to cell lysis via bead beating for 5 min. The cell suspension was centrifuged for 5 min at 15,000 *g*, and 100 µL of the supernatant was transferred into a fresh tube containing 800 µL of resuspension buffer. The mixture was then prewarmed at 37°C for 10 min. The substrate *p*-nitrophenyl-β-D-glucuronide was resuspended in resuspension buffer (10 mM), and 100 µL of the substrate was added into the mixture and incubated at 37°C for 30 min. The enzymatic reaction was stopped by adding 500 µL of 1 M sodium bicarbonate, and the absorbance of the solution was measured at 405 nm (Abs_405_). The β-glucuronidase activity was determined as Abs_405_/OD_600_.

### L2 fibroblasts plaque assay

Plaque assays were performed as previously described (33). Briefly, murine L2 fibroblast cells were maintained in Dulbecco’s Modified Eagle Medium (DMEM) and grown to confluency in 6-well microtiter plates. Subsequently, bacterial cells were grown overnight in BHI at 37°C under static conditions. The OD_600_ of the culture was measured and normalized to an approximate OD_600_ of 0.7 and washed twice with PBS. The washed cell suspension was diluted 5×, and 20 µL of this diluted suspension was used to infect each well for each strain. Each experiment was performed with four technical replicates. After an hour of incubation at 37°C under 5% CO_2_ conditions, the medium was removed, and L2 fibroblast monolayers were washed once with pre-warmed PBS followed by the addition of 2 mL fresh DMEM supplemented with 10 µg mL^−1^ of gentamicin in 0.7% agarose to the cells. The plates were incubated at 37°C under 5% CO_2_ condition for 3 days, after which Neutral red (Sigma-Aldrich) was added to facilitate easier visualization and enumeration of the plaques using a micrometer (Finescale, Orange County, CA, USA).

### Intracellular growth and fluorescent staining in PtK2 cells

The intracellular growth capacity of *Lm* and its variants was determined in Potoroo tridactylis kidney (PtK2) epithelial cells as previously described (30). Briefly, PtK2 cells were grown on sterile coverslips till confluency, and bacterial cells were infected at an MOI of 1:100 (cells:bacteria). After an hour of incubation at 37°C under 5% CO_2_ condition, the medium was removed and washed with pre-warmed PBS twice. Subsequently, 1 mL fresh DMEM medium supplemented with 10 µg mL^−1^ of gentamicin was added back to the dishes containing the coverslips, and the dishes were incubated at 37°C under 5% CO_2_ conditions. Coverslips were removed at appropriate time intervals and vortexed in 1 mL of sterile water to induce lysis of Ptk2 cells, and the supernatant was serially diluted and spot plated onto BHI plates for the enumeration of intracellular bacterial cells.

Fluorescent staining was also performed to visualize the colocalization of bacterial cells with actin during the infection process. Infection of PtK2 cells was carried out as described above with an MOI of 1:40 and at 24 h post-infection, coverslips were removed, and fixed in PBS with 0.4%, vol/vol paraformaldehyde. Subsequently, coverslips were treated with PBS with 0.1%, vol/vol Triton-X for 10 min, followed by a blocking step with PBS with 10%, wt/vol BSA for 10 min. After blocking, the coverslip was dipped in sterile PBS 5×, and primary antibody against *Listeria monocytogenes* (Abcam; cat. No: AB35132) was applied at a final concentration of 8 µg mL^−1^ for 20 min. Subsequently, the coverslips were dipped in sterile PBS 5×, and secondary antibody (α-rabbit) conjugated with a fluorophore (Invitrogen; cat. No: A11011) was applied at a final concentration of 4 µg mL^−1^, as well as the fluorescent stain for F-actin (Invitrogen; cat. No: R37110) for 20 min. After incubation, the coverslips were dipped in sterile PBS 5× and mounted onto a slide using a mounting medium containing DAPI (Vectashield; cat. No: H-1500). The cells on the coverslips were visualized using a confocal laser microscope (Zeiss LSM-900) with a Plan Apochromat 63 ×  1.4 numerical aperture oil objective. A 561-nm laser was used to excite Texas Red, a 488-nm argon laser was used to excite phalloidin, and 405-nm laser to excite DAPI.

### Statistical methods

Statistical analyses were performed with Prism 10 (GraphPad software) using paired Student’s *t*-test.

## RESULTS

### The EIIB^Gat^ domain of Type-I Mga harbors a novel motif with potential for cysteine phosphorylation

We have previously demonstrated that in *S. pyogenes* NZ131 (GAS) the Rgg2/3 quorum-sensing system is subject to carbon catabolite repression with the induction of QS occurring when cells were grown in mannose (14). CCR of the QS system is mediated by both Mga, the master regulator of virulence, and ManL, the EIIA/B subunit of the PTS^Man^ (15); however, the mechanism by which these two components converge to provide CCR of the QS system remains to be elucidated. Mga is a transcriptional regulator that exists as two allelic isoforms within the GAS population. The two isoforms (Type I and Type II) are nearly identical, differing only at the extreme C-terminus end, which has no described function thus far (34).

It was previously shown that the PRD1 and PRD2 of Mga are phosphorylated by HPr-His15~P, which impacts Mga’s ability to oligomerize (7). Further analysis revealed that the EIIB^Gat^ domain of Mga is essential for oligomerization (34). Prior studies characterizing the EIIB^Gat^ domain of Mga have been performed with Mga of Type-I lineage, in which the authors reported that the conserved cysteine found in MtlR and ManR EIIB^Gat^ domains is absent, being instead replaced by a glutamine (34). However, an alignment of the amino acid sequence of the Mga of both Type-I and Type-II lineages revealed the presence of the EIIB^Gat^ cysteine at the same position as found in MtlR and ManR. Realignment of the amino acid sequence of the EIIB^Gat^ domain of both Type-I and Type II Mga against EIIB subunits from different PTS families, as well as against the EIIB^Gat^ domains from well-studied transcriptional regulators that harbor these domains (Table S1), confirmed that in Type-I Mga an alanine, is present in place of cysteine. However, we noted that the subunits that contain the cysteine residue are surrounded by highly conserved amino acid sequences, depending on the PTS family (Fig. 1). Interestingly, there is no observable conservation of these residues between the EIIB^Gat^ of Type-II Mga and EIIB subunits of PTS components or EIIB^Gat^ domains of MtlR, LicR, and ManR. Instead, the cysteine residue in Type-II Mga is gated by a triad of aromatic amino acids (YCFF) bearing resemblance to a motif (YHFF) that is uniquely found in glycerol kinases of Gram-positive bacterial species (35). The histidine residue within this glycerol kinase motif is phosphorylated by HPr-His15~P to enhance its enzymatic activity (35–37). This suggested the possibility of a regulatory role imparted by the phosphorylation of the cysteine residue within the novel Type-II Mga motif in the EIIB^Gat^ domain, possibly mediated via PTS components.

**Fig 2:**
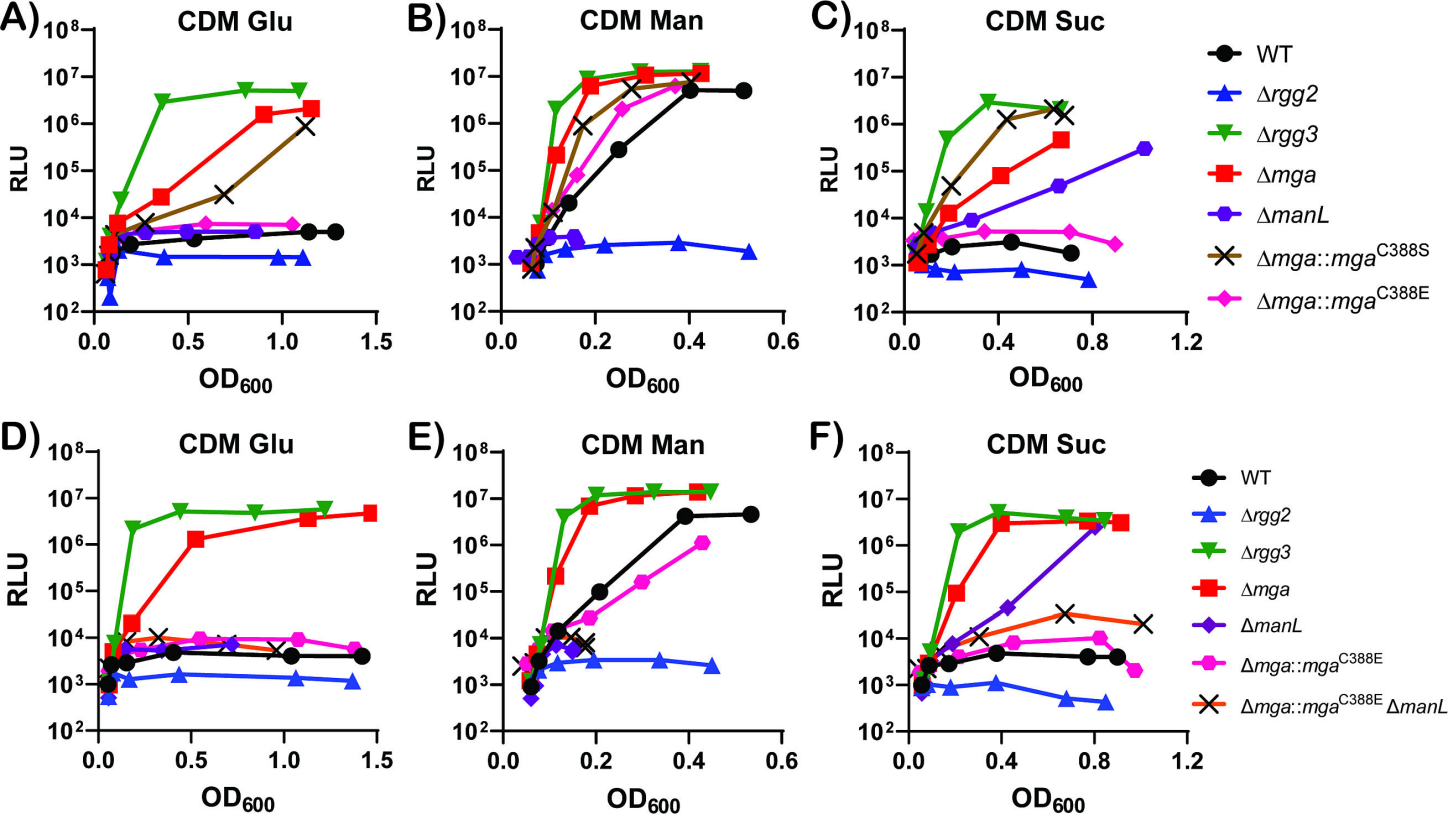
The cysteine residue in Type II Mga harbors a regulatory role in modulating the Rgg2/3 QS system during growth in different carbohydrates. Induction of the Rgg2/3 QS circuitry is monitored by the expression of luciferase genes driven by the promoter of the pheromone encoding gene, *shp3* in chemically defined media supplemented with glucose (**A and D**), mannose (**B and E**), and sucrose (**C and E**). RLUs were obtained by normalizing the abundance of luminescence units per second to the OD_600_ of the corresponding sample and plotted on the *Y* axis on a logarithmic scale, while the *X* axis is the OD_600_ value of the sample. Results shown are representative of at least three biological replicates.

To investigate the possibility that the Mga EIIB^Gat^ domain cysteine residue may be a target for phosphorylation and thereby regulate the activity of Mga, a serine (C388S) or glutamic acid (C388E) substitution was introduced to simulate a phospho-ablative or phospho-mimetic substitution, respectively. The induction of Rgg2/3-dependent QS was then assessed following bacterial growth in the presence of permissive (mannose) and non-permissive (glucose and sucrose) carbohydrates (Fig. 2). The Mga C388S mutant was found to resemble a Δ*mga* mutant in that Rgg2/3 QS was constitutively induced regardless of the carbohydrate used for growth, suggesting that either the serine substitution of cysteine resulted in a non-functional protein or the serine substitution mimicked an inactive state. In contrast, the Mga C388E mutant resembled WT strains in that Rgg2/3 QS was suppressed during growth in both glucose or sucrose and induced during growth in mannose, suggesting that the C388E mutation supports a fully functional Mga. Based on the phospho-ablative and phosphor-mimetic substitution results, it is possible that the cysteine residue of the Type-II Mga EIIB^Gat^ domain may indeed be phosphorylated and act in combination with the phosphorylation of the PRD1 and/or PRD2 domain to mediate the carbohydrate-dependent regulation of the Rgg2/3 QS system.

### The cysteine residue within the Mga EIIB^Gat^ domain YCFF motif may be phosphorylated by EIIA/B of PTS^Man^

We previously reported that ManL, the EIIA/B component of PTS^Man^, plays a role in repressing Rgg2/3 QS during growth in select carbohydrates such as sucrose [(15) and Fig. 2], but the actual mechanism by which this repression occurs is not known. Based on examples of other PRD regulators harboring EIIB domains, phosphorylation of the conserved cysteine residue alters their activity and that phosphorylation is typically mediated through the EIIA of their cognate PTS systems (3, 5, 38). We therefore speculated that the GAS Type-II Mga EIIB^Gat^ cysteine may be phosphorylated by ManL to regulate Rgg2/3 QS in a carbohydrate-specific manner. To test this hypothesis, a Δ*manL* deletion was constructed in the Mga C388E background and the mutant’s ability to induce Rgg2/3 QS was assessed following growth in the presence of different carbohydrates. When grown in glucose, Rgg2/3-mediated QS remained repressed in the Mga C388E Δ*manL* mutant; the mutant was unable to grow in mannose due to the absence of a functional PTS^Man^. However, following growth in sucrose, Rgg2/3 QS remained repressed in the Mga C388E Δ*manL* mutant in contrast to the Δ*manL* mutant alone, in which QS was induced. Collectively, these results suggest that ManL could potentially be the phospho-donor of the cysteine residue in the EIIB^Gat^ domain of Mga and that the phosphorylation of EIIB^Gat^ on this cysteine is responsible for repressing the Rgg2/3 QS system during growth in PTS-dependent non-glucose/mannose carbohydrates.

### The novel YCFF motif is conserved in transcriptional regulators of several Gram-positive pathogens

**Fig 3:**
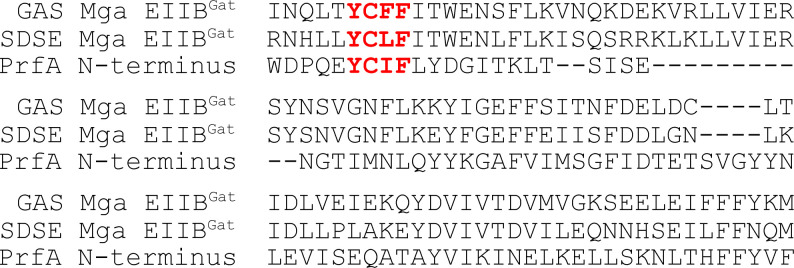
Amino acid sequence alignment of Mga and PrfA reveals the conservation of this novel motif. The amino acid sequence alignment of Type II Mga EIIB^Gat^ domain (382–477 a.a) from GAS, Mga EIIB^Gat^ domain (385–479 a.a) from SDSE and the N-terminus of PrfA (32–117 a.a). Highlighted in red is the presence and near conservation of this Aro-Cys-(Aro/Xle)-Aro motif between Mga and PrfA.

To extend these functional analyses, we investigated whether the YCFF motif appears unique to Mga or if it is present in other transcriptional regulators. We found that the Mga homologs of *Streptococcus canis* and *Streptococcus dysgalactiae* subsp. *equismilis* (SDSE) harbor a similar but slightly varied motif, such that the amino acid after the cysteine residue of the motif is a hydrophobic branched-chain amino acid instead of an aromatic amino acid (Fig. 3). A similar degenerate motif is also found in the MtlR homolog of *Enterococcus faecalis* V583 (EF0407; motif: FCIF), suggesting that this motif could potentially have a modulatory function in transcriptional regulators that assist in adjusting gene expression during growth in the presence of different carbohydrates. These observations also provided a potential consensus for this motif, presenting it as Aro-Cys-(Aro/Xle)-Aro [Aro; aromatic and Xle; leucine or isoleucine].

During a non-exhaustive curation of transcriptional regulators that possess a similar four amino acid motif, we serendipitously discovered a well-studied transcriptional regulator that does not harbor any PRDs but does possess a degenerate Aro-Cys-(Aro/Xle)-Aro motif [YCIF like Mga of *S. canis* and SDSE (YCLF)]. This transcriptional regulator is the master regulator of virulence known as PrfA in *L. monocytogenes*, belonging to the Crp/Fnr family of transcriptional regulators (Fig. 3). It has been well-documented that PrfA-dependent expression of virulence factors in *Lm* is subjected to CCR in a CcpA-independent manner (17, 39). It appears that CCR is conferred through the inhibition of PrfA activity (40), and this repression is mediated by PTS components, specifically the phosphorylation of EIIA and/or EIIB subunits (22). Further analyses have proposed that the inducible mannose PTS system, PTS^mpt^, which is homologous to the ManLMN system of GAS, is the PTS that confers CCR on PrfA activity (21, 41). The mechanism by which PTS^mpt^ regulates the activity of PrfA during growth in the presence of different carbohydrates remains to be determined. We, therefore, decided to explore whether the presence of the PrfA YCIF motif might play a role in the regulation of PrfA activity.

### The cysteine residue within the PrfA YCIF motif may serve a regulatory function

**Fig 4:**
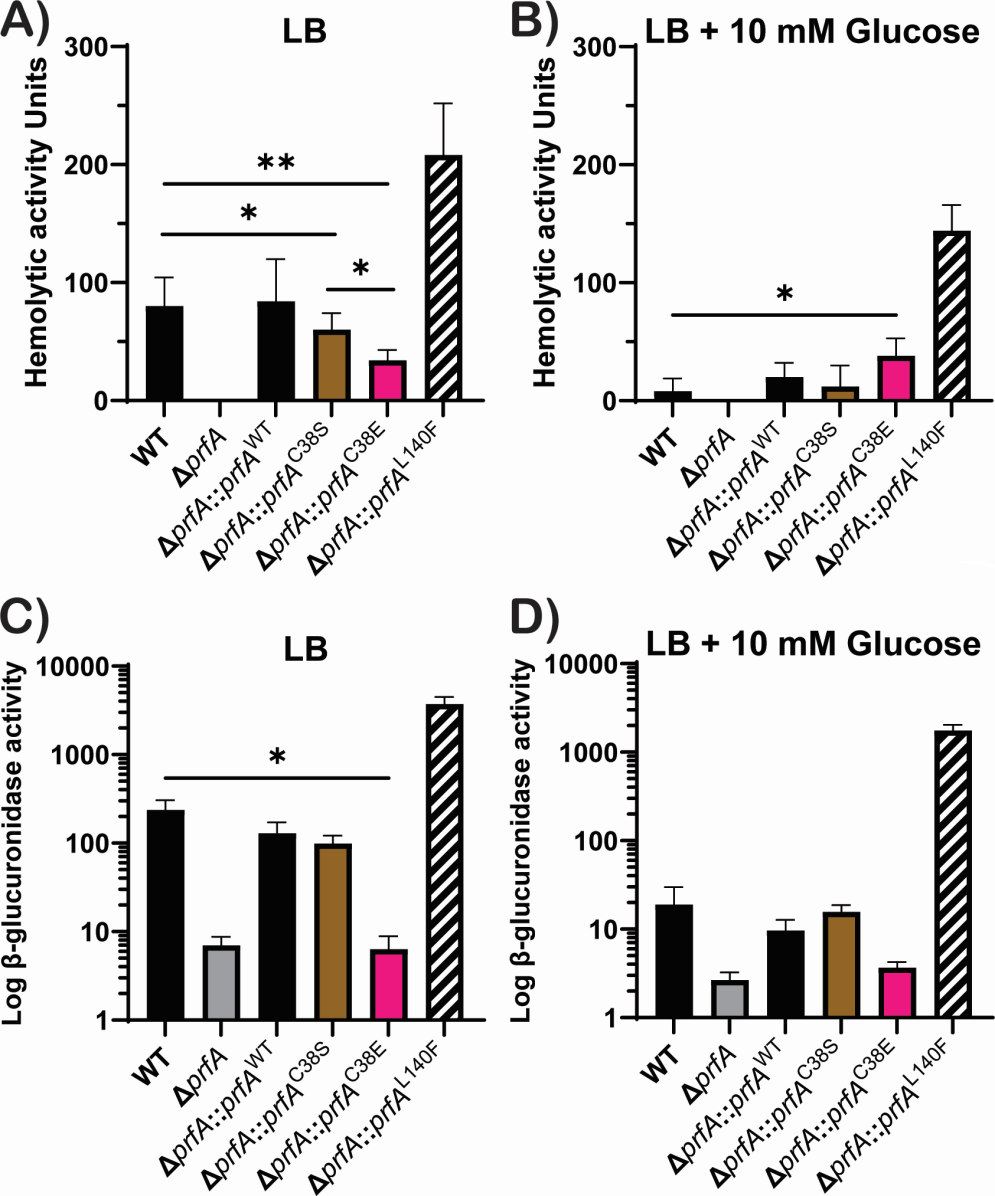
The phosphomimetic variant of the cysteine residue in the novel motif of PrfA showed dysregulation of virulence factors. Cells were grown to the stationary phase in LB or LB supplemented with 10 mM glucose and (A and B) the supernatant was used to determine the amount of hemolysin secreted by each strain, while (C and D) whole cell lysate was used to determine the amount of β-glucuronidase, which is a proxy for *actA* expression. The results shown is the mean of at least three biological replicates ± SD. **P < 0.001 and *P < 0.05.

To explore if the YCIF motif, specifically the potential phosphorylation of the cysteine residue, might impact PrfA activity, we adopted the same strategy of mutating the cysteine residue to serine (PrfA C38S) and glutamic acid (PrfA C38E) to simulate a phospho-ablative and phospho-mimetic conformation, respectively. Virulence-associated phenotypes of these variants in comparison to WT and the Δ*prfA* mutant were then assessed. The secretion of the hemolysin LLO, encoded by *hly* and whose expression is dependent on PrfA, was measured based on hemolytic activity present in supernatants following bacterial growth in broth culture (Fig. 4A and B). Both WT and Δ*prfA* mutant strains complemented with the WT allele of *prfA* (Δ*prfA::prfA*^WT^) secreted abundant LLO-dependent hemolytic activity when grown in LB broth media, while the Δ*prfA* mutant exhibited no detectable hemolytic activity (Fig. 4A). As expected, introduction of the constitutively active *prfA* L140F (PrfA*) allele resulted in significantly increased levels of hemolytic activity in comparison to WT or the Δ*prfA* complemented with the wild-type *prfA* allele. The strain variant that contained a serine substitution of the cysteine within the YCIF motif of PrfA (Δ*prfA::prfA* C38S) was observed to secrete a slightly lower level of hemolytic activity (60 ± 14.1 units) than that observed for WT (80 ± 24.5 units) grown in LB (*P* = 0.0341), suggesting that the C38S substitution modestly reduced the ability of PrfA to induce *hly* expression. The strain variant harboring the glutamic acid substitution of the cysteine (Δ*prfA::prfA* C38E) exhibited a more significant reduction in hemolytic activity, with levels that were approximately twofold lower (34 ± 8.944 units) than the levels observed for WT. The PrfA C38E substitution, therefore, has a negative impact on PrfA activity with respect to the induction of *hly* expression. To further characterize the potential effect of the PrfA cysteine substitutions on PrfA activity under conditions of CCR, hemolytic activity was assessed following bacterial growth in LB supplemented with 10 mM glucose and 50 mM MOPS pH 7.2 (Fig. 4B). As anticipated, PrfA-dependent hemolytic activity was reduced in the presence of glucose and was extremely low for both WT and Δ*prfA::prfA*^WT^, while glucose had minimal effect on PrfA*-dependent *hly* expression based on activity as significant lysis of red blood cells was still observed. The strain complemented with the *prfA* C38S also exhibited reduced lysis of red blood cells when grown in media containing glucose in comparison to no glucose, indicating that the mutant was still subject to CCR (Fig. 4B). Interestingly, the PrfA C38E variant strain exhibited no significant change in hemolytic activity when grown in LB supplemented with 10 mM glucose (*P* = 0.0179); its consistent level of activity was approximately fivefold higher than the activity of WT with glucose, suggesting that the PrfA C38E variant is insensitive to CCR.

We next examined the expression of another PrfA-dependent virulence factor, *actA*, using a β-glucuronidase reporter gene fusion (*gus*) transcriptionally linked to *actA* expression within its native locus (42). Cells were grown in either LB or MOPS-buffered LB supplemented with 10 mM glucose and subsequently lysed to determine the amount of β-glucuronidase produced as a readout of *actA* expression (Fig. 4C and D). WT, the *prfA*-complemented Δ*prfA* mutant, and prfA*-complemented Δ*prfA* mutant all exhibited moderate to high levels of β-glucuronidase activity, whereas low levels of β-glucuronidase activity were observed for Δ*prfA* strains, confirming anticipated expression patterns based on previous characterization of these *prfA* mutants (42–44). Strains containing the PrfA C38S variant exhibited β-glucuronidase activity at levels similar to those of WT, whereas the PrfA C38E variant resembled the Δ*prfA* mutant strain with very low levels of *actA* expression. The addition of glucose to the media dramatically lowered GUS activity in all but the Δ*prfA, prfA C38E*, and prfA* strains, indicating that these strains were less sensitive to catabolite repression.

**Fig 5:**
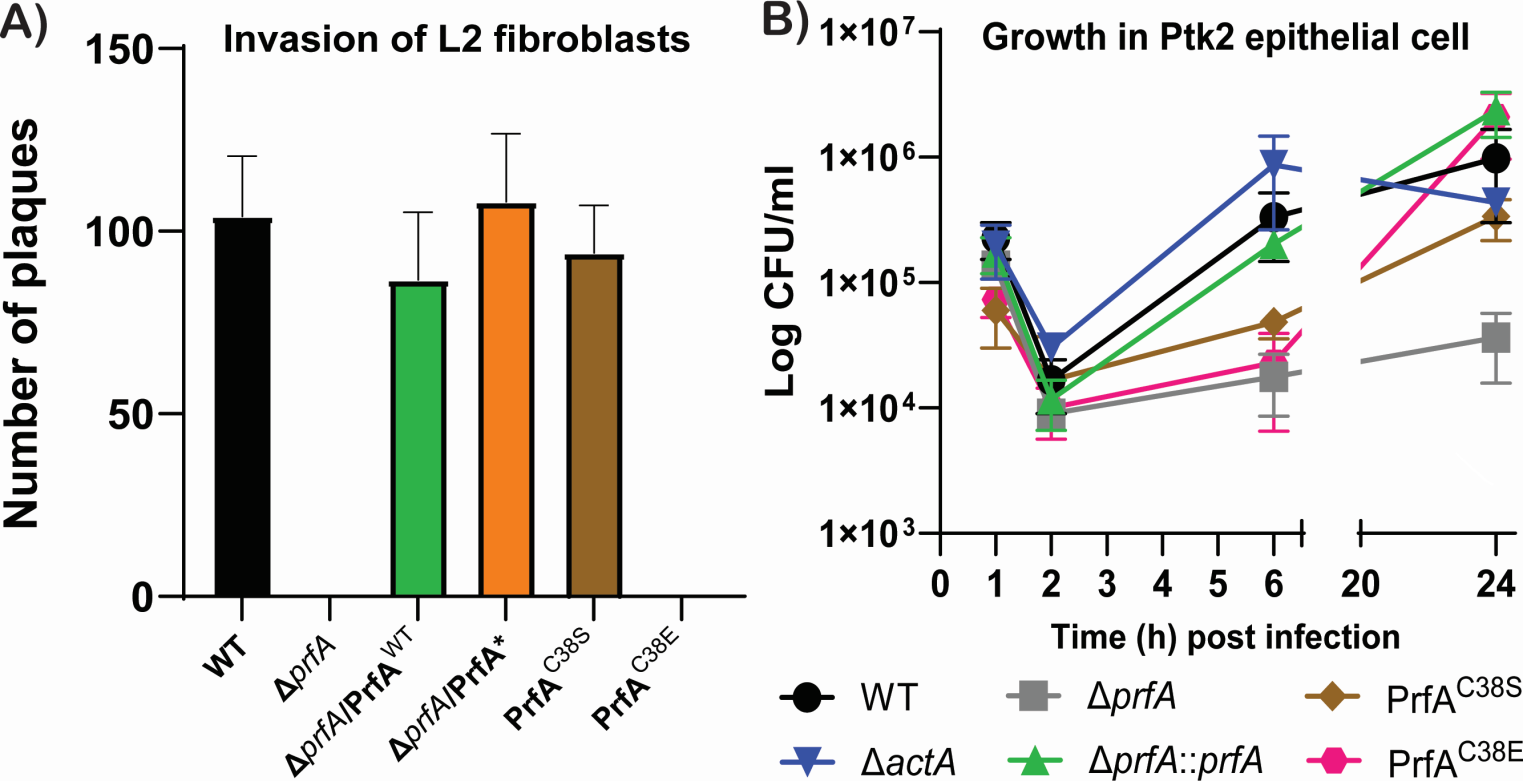
The phosphomimetic variant of the cysteine residue in the novel motif of PrfA is attenuated in virulence *in vitro*. *L. monocytogenes* WT and their respective variants were used to determine their plaque-forming capabilities in (A) L2 fibroblast cells and (B) intracellular growth capabilities in Ptk2 cells. Cells were infected with the respective samples at an MOI of 1:100. For plaque assay, plaques were stained with neutral red 3 dpi for easier visualization and enumeration. For intracellular growth in Ptk2 cells, coverslips (*n* = 3) were removed and resuspended in 1 mL of sterile water, serially diluted, and spot plated (10 µL) onto BHI plates to enumerate colony-forming units (CFUs). Each experiment was performed at least three times, and the results shown are representative of one experiment.

Optimal induction of PrfA activity takes place within the cytosol of infected host cells, where PrfA activation is required for *actA* expression and actin-based bacterial motility and spread (42). To better assess the activity of the PrfA cysteine substitution mutants within the context of host cell infection, we examined the ability of strains containing PrfA C38S and PrfA C38E to infect, replicate, and spread based on the formation of zones of clearing or plaques within monolayers of L2 fibroblast cells (Fig. 5A). As anticipated, WT *Lm* formed distinct plaques within infected L2 monolayers, whereas the Δ*prfA* mutant failed to form any visible plaques. Complementation of the Δ*prfA* mutant with either a wild-type *prfA* allele or the activated prfA* allele formed plaques with the same size and frequency as WT-infected cells. *Lm* containing the PrfA C38S variant, which appears to exhibit nearly WT patterns of virulence factor secretion *in vitro* (Fig. 5), also formed plaques of comparable size and frequency to that of WT. The PrfA C38E variant resembled the Δ*prfA* mutant in that it formed no visible plaques, indicating a significant defect in cellular invasion and/or intracellular growth and spread (Fig. 5A).

The absence of visible plaques for the *Lm* PrfA C38E mutant could reflect a complete absence of PrfA activity within infected cells, analogous to a Δ*prfA* mutant that is trapped within host cell vacuoles and cannot reach the cytosol where bacterial replication occurs. Alternatively, *prfA* mutants have been previously described that possess sufficient PrfA activity with low levels of LLO to lyse the vacuole, but insufficient PrfA activity to induce *actA* expression once the bacteria reach the cytosol (PrfA Y154C mutants) (45). *Lm* strains containing the *prfA* Y154C mutation also fail to form visible plaques in L2 monolayers, although they are capable of intracellular bacterial replication (45). To determine if the PrfA C38E mutant more closely resembled a complete loss of PrfA function or alternatively, a PrfA mutant capable of vacuole escape but incapable of actin polymerization, we infected Potoroo tridactylis kidney (Ptk2) epithelial cells and examined bacterial intracellular growth (Fig. 5B). For comparison, we included the Δ*actA* mutant, which is able to invade, escape the vacuole, and replicate within mammalian cells but is unable to associate with host cell actin machinery and thus does not spread to adjacent cells (46). PtK2 cells infected with wild-type *Lm* or the Δ*actA* mutant exhibited robust intracellular bacterial replication over time as indicated by the increase in recovered bacterial CFU. In contrast, the Δ*prfA* mutant, which remains trapped within host cell vacuoles (47), exhibited no significant bacterial replication based on recovered CFU; however, the introduction of the wild-type *prfA* allele was sufficient to restore replication (Fig. 5B). Interestingly, cells infected with the PrfA C38S and PrfA C38E strains exhibited little to no increase in CFU at 6 h post-infection in comparison to cells infected with the WT, *prfA*-complemented Δ*prfA* mutant, or Δ*actA*; however, both *prfA* variants exhibited evidence of intracellular replication based on recovered CFU by 24 h post-infection, reaching CFU levels similar to those of cells infected with WT *Lm*. These results suggest that strains containing PrfA C38S or PrfA C38E may be delayed for vacuole escape; however, the bacteria appeared capable of eventually reaching the host cytosol as bacterial replication does occur.

**Fig 6:**
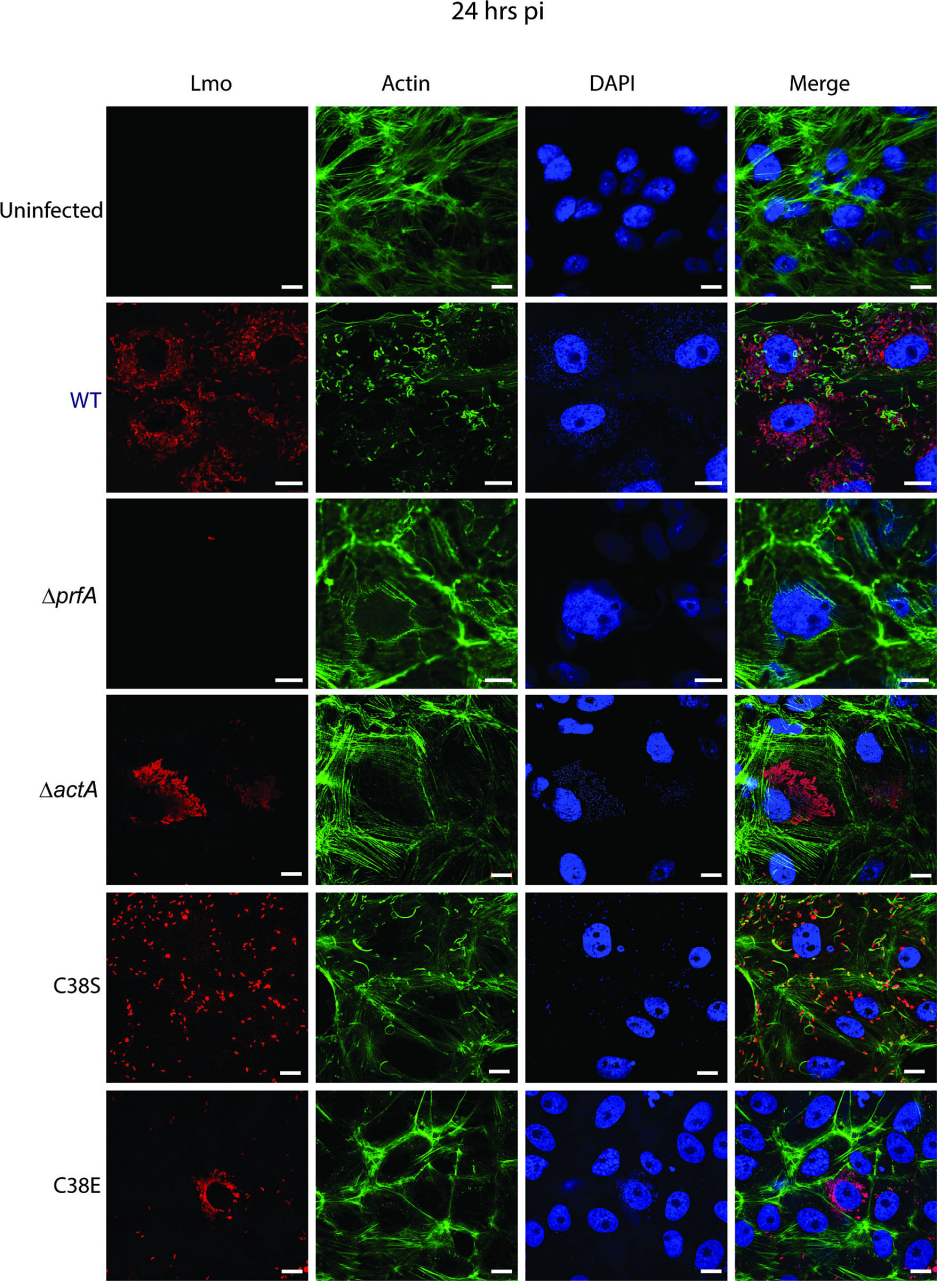
The phosphomimetic variant of the cysteine residue in the novel motif of PrfA showed a cell-cell spreading defect. *L. monocytogenes* WT and their following mutants: Δ*prfA*, Δ*actA*, C38S, or C38E were used to infect PTK2 cells at an MOI of 1:40. Cells were fixed after 24 h p.i. and subsequently probed with anti-Lm antibody, followed by Texas Red-conjugated secondary antibody, phalloidin to stain actin (green), or DAPI to stain nuclei (blue). Red channel: *L. monocytogenes* (LMO), green channel: actin, and blue channel: DNA. Scale bar = 10 µm.

Given that the PrfA C38E mutant failed to form plaques in L2 fibroblast monolayers and yet was capable of intracellular replication—a facet of infection that requires bacterial escape from host cell vacuoles—we speculated that this mutant failed to associate with host cell actin and spread cell-to-cell. We therefore examined infected PtK2 cells at 24 h post-infection using fluorescence-based microscopy and staining for *Lm* and filamentous actin (Fig. 6). Consistent with the results shown in Fig. 5B, the PrfA C38E mutant was able to proliferate to high CFU density within infected Ptk2 cells, similar to the Δ*actA* mutant, and like the Δ*actA* mutant failed to associate with host cell actin (Fig. 6; Table 1). In contrast, the PrfA C38S variant was associated with host cell actin and was able to spread through confluent monolayers of Ptk2 cells, indicating that this mutant retained the ability to express *actA*, polymerize actin, and spread cell-to-cell. Taken together, these results indicate that strains containing the PrfA C38S phospho-ablative mutation retained PrfA function and were capable of intracellular bacterial growth and cell-to-cell spread. In contrast, mutant strains containing the PrfA C38E phospho-mimetic mutation exhibited defects in actin association and actin-based motility, a phenotype consistent with a failure of PrfA C38E to become fully activated within the cytosol of infected host cells. These results suggest a model in which the potential phosphorylation of PrfA at C38 relieves glucose repression of PrfA activity but prevents the full activation of PrfA necessary to support actin-based motility and cell-to-cell spread.

**TABLE 1:** Percentage of cells associated with actin during infection of Ptk2 epithelial cells

	FOV 1	FOV 2	FOV 3	FOV 4	FOV5	FOV 6	Average
WT (%)	75.44	72.09	71.43	69.57	67.95	64.56	70.17
Δ*prfA*	0	0	0	0	0	0	0
Δ*actA*	0	0	0	0	0	0	0
PrfA^C38S^ (%)	50	63.33	67.53	66.66	66.66	67.64	63.64
PrfA^C38E^	0	0	0	0	0	0	0

## DISCUSSION

The idea that bacterial pathogens may sense their location in the host at least partially based on what’s available to eat has been previously proposed (48, 49). Available carbon sources may serve as an environmental trigger, and indeed the expression of virulence genes has been linked in several bacterial pathogens to metabolic pathways and their regulation (24, 50, 51). CCR has been previously shown to influence the activity of transcriptional activators that regulate virulence gene expression through complex mechanisms (7, 21, 52). Here, we provide another potential mechanism by which carbon metabolism may be linked to virulence for two important bacterial pathogens, GAS and *Lm*. Our data suggest that conserved cysteines within a potentially novel motif that is shared between GAS Mga and *Lm* PrfA may serve as targets for phosphorylation that in turn links carbon metabolism to Mga and PrfA activity. The use of phosphomimetic and phosphoablative substitutions of the cysteine supports this hypothesis; however, confirmation awaits direct demonstration of cysteine phosphorylation and the subsequent changes in regulator activity.

The identification of the Aro-Cys-(Aro/Xle)-Aro motif within the EIIB^Gat^ domain of Mga provides a potential mechanistic link between Mga, ManL of the PTS^Man^ system, and CCR of the Rgg2/3 QS system of GAS. We have previously characterized the environmental signals that influence the Rgg2/3 QS system in *S. pyogenes* NZ131 (M49 serotype; Type II Mga allele) and discovered that Rgg2/3 system is only induced when cells are grown in mannose while remaining repressed in either glucose or sucrose as sole carbon sources, indicating that the QS system is under CCR (14, 15). We further determined that the CCR of the QS is mediated through a CcpA-independent pathway that requires the EIIA/B component, ManL of PTS^Man^, and the master regulator of virulence, Mga (15). Mga contains an EIIB^Gat^ domain, and the phosphorylation of a conserved cysteine within this domain in other transcriptional regulators, such as MtlR of *Bacillus subtilis* (5), ManR of *L. monocytogenes* (38), and CelR of *S. pneumoniae* (53) has been shown to regulate the activity of these transcriptional regulators. The results obtained here using cysteine phosphomimetic and phosphoablative substitutions are consistent with the premise that the activity of Type-II Mga could also be regulated through the phosphorylation of the conserved cysteine residue within its EIIB^Gat^ domain. It is possible that the phosphorylation of the Mga cysteine induces a conformational change of Mga that results in its oligomerization, as the Mga EIIB^Gat^ domain has been previously associated with Mga oligomerization (34). Alternatively, the modulation of Mga could be even more complex, such that in addition to the combinatorial phosphorylation of both PRDs and the EIIB^Gat^ domain, regulation could also involve protein-protein interactions via the Mga EIIB^Gat^ domain with ManL, as observed for both MtlR and ManR (3, 54).

The Aro-Cys-(Aro/Xle)-Aro motif was also identified in PrfA of *Lm*. It has been recognized for decades that PrfA-dependent expression of virulence genes is subjected to CCR (39). For example, growth in glucose results in high intracellular levels of P-Ser46-HPr (24), and the lack of phosphorylated EIIA and EIIB components has been associated with reduced PrfA-dependent gene expression. *Lm* relies on two PTS systems to sense and exert CCR; the primary system is the constitutively expressed PTS^mpo^, which is an inefficient transporter but instead acts as a sensory unit for glucose/mannose. The second PTS system is the ManR-inducible PTS^mpt^, which is highly efficient for the transport of glucose and mannose (41, 55). The presence of phosphorylated EIIAB^mpt^ has been associated with CCR of PrfA (21, 56). The question remains as to how PrfA receives the signal corresponding to available carbohydrate for the regulation of its activity. Herro et al. (24) has proposed that the CCR could potentially be mediated through the phosphorylation of PrfA by EII components. Based on our work with phosphomimetic and phosphoablative cysteine substitutions, we suggest that EIIAB^mpt^ could potentially be the phospho-donor with phosphorylation occurring at the PrfA Cys-38 residue. The formal confirmation of PrfA Cys-38 and Mga Cys-388 as phosphorylation sites is challenging given the extremely labile nature of phospho-cysteine (57, 58).

Based on the protein structure of PrfA (PDB: 1OMI), the cysteine residue within the YCIF motif appears surface-exposed and is located near the connective junction between the N-terminus β-jelly roll domain and the long α-helix linker, which is the binding site of glutathione (GSH) (59). The phospho-ablative serine substitution (PrfA C38S) did not dramatically impact the activity of PrfA, while the phospho-mimetic glutamic acid substitution (PrfA C38E) rendered the mutant protein less susceptible to CCR but apparently unable to become fully activated within the host cell cytosol. We have previously identified a mutation, PrfA Y154C, that maintained the ability to induce sufficient LLO expression for vacuole escape but which, similar to PrfA C38E, was defective for *actA* induction in the cytosol and hence intracellular bacterial motility (45). We speculated that the Y154C mutation may prevent the conformational change required for full PrfA activation following the entry of the bacteria into the cytosol. It is possible that the PrfA C38E mutant imposes a similar constraint on PrfA activation or alternatively it might interfere with GSH co-factor binding and thereby reduce the ability of PrfA to activate target gene expression. However, the PrfA C38S mutation retained actin-based motility and the ability to spread within cell monolayers. We thus favor the hypothesis that the phosphorylation of PrfA cysteine 38 serves to regulate PrfA activity in the presence of PTS sugars, such as glucose, preventing full PrfA activation until *Lm* enters the cytosol and switches to the use of non-PTS sugars, such as glycerol or glucose-6-phosphate.

**Fig 7:**
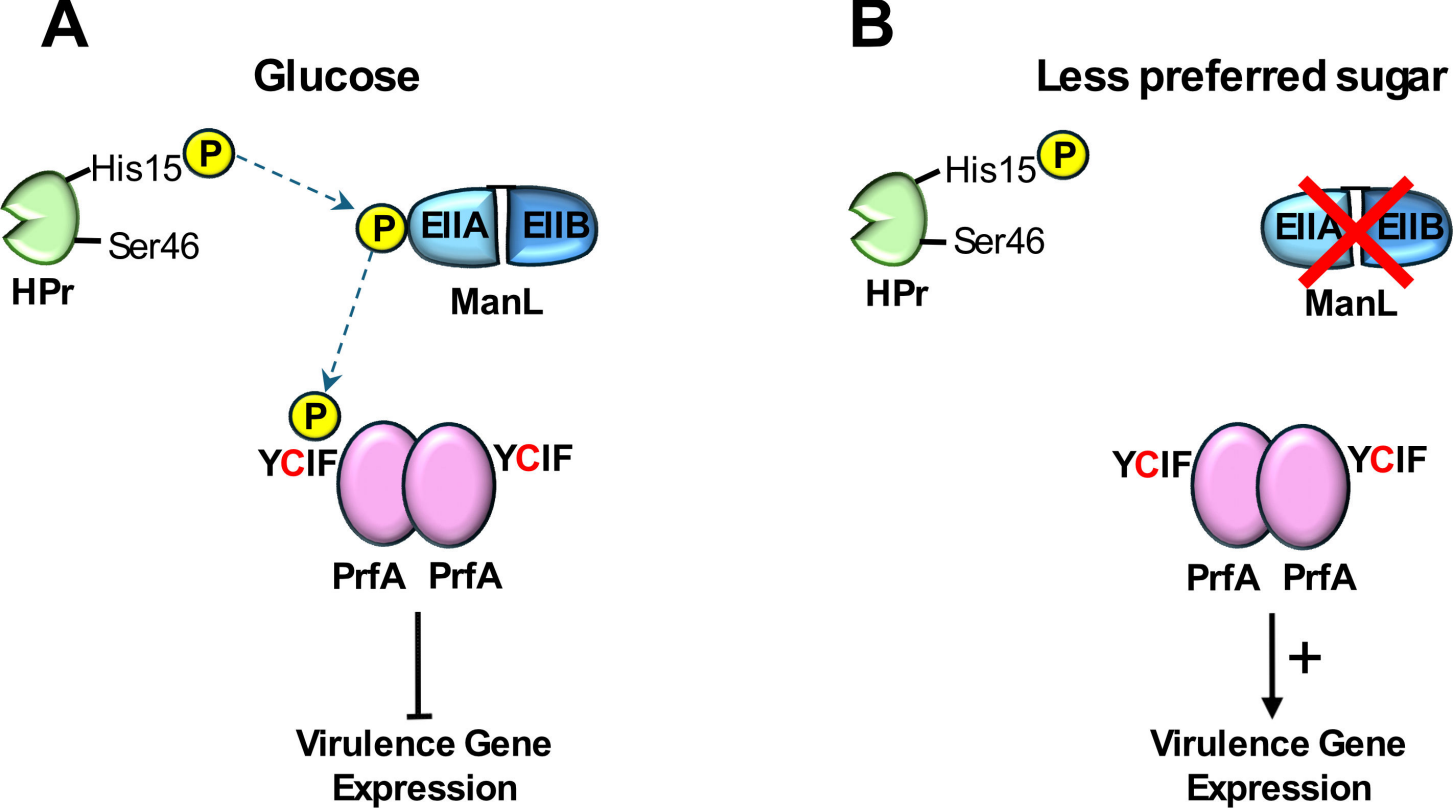
A proposed model theorizing how the novel motif impacts the activity of PrfA in *Listeria monocytogenes*. Demarcated lines indicate the flow of phosphate (yellow circle). (**A**) In *Lm*, the expression of virulence factors is repressed when cells are grown in glucose, due to the inactivation of PrfA, possibly through the phosphorylation of the cysteine residue within this novel motif by ManL, the EIIA/B subunit of PTS^mpt^. (**B**) Upon growth in less-preferred carbon source, such as glucose-6-phosphate, ManL expression is suppressed and consequently the lack of phosphorylation of the cysteine residue results in the activation of PrfA, therefore upregulating the expression of virulence genes.

Based on all the findings reported here, we propose a model specific to *Lm* to describe how the cysteine-containing motif within PrfA potentially mediates signal transduction via cysteine phosphorylation by PTS components (Fig. 7). The phosphorylation of the PrfA YCIF motif can be integrated into the model proposed by Aké et al. (21) to describe how the constitutively expressed PTS^mpo^ senses glucose and modulates the activity of the regulator ManR to induce the expression of the inducible PTS^mpt^ to facilitate the import of glucose. During *Lm* growth in glucose-replete conditions, the EIIA/B subunit of either PTS^mpt^ (ManL) or PTS^mpo^ is phosphorylated for the transport of glucose and, in addition, phosphorylates PrfA at its YCIF motif to inhibit PrfA activity in response to glucose (Fig. 7A). Phosphorylation of the cysteine could lead to a conformational change to prevent the binding of GSH within the PrfA tunnel or structurally inhibit full PrfA activation, thus preventing the full induction of the PrfA regulon. PrfA is known to exist as a dimer, thus it is possible that the phosphorylation of one or both cysteines may occur and/or be needed to influence activity. In the absence of carbohydrates that rely on PTS^mpt^ for transport, ManR will be rendered inactive and subsequently shut down the expression of PTS^mpt^. The reduced availability of ManL will reduce PrfA YCIF cysteine phosphorylation, allowing the binding of GSH to PrfA and/or the conformational change required for full PrfA activation (Fig. 7B).

For GAS, the influence of phosphorylation on Mga activity appears more complex, given the presence of the phosphorylation sites within the two PRD domains together with the cysteine within the EIIB^Gat^ domain. During growth in glucose-replete conditions, Mga is active and Mga-dependent virulence genes are expressed. HPr-His15~P phosphorylates the PRDs of Mga and ManL, and as the PTS^Man^ system is not required for the import of glucose, we propose that phosphorylated ManL will then phosphorylate Mga on the cysteine residue within the EIIB^Gat^ domain to initiate CCR of Rgg2/3 QS through combinatorial phosphorylation of PRDs and the cysteine within the EIIB^Gat^ of Mga (Fig. S2). During growth in glucose-deplete conditions where another non-mannose substrate is available (sucrose for example), CCR is relieved as HPr-His15~P is able to charge all available EIIA subunits, priming them to be available for the transport of any available carbohydrate to facilitate growth (26, 27). Under these conditions, we predict that the PRD domains will not be phosphorylated but that the phosphorylated ManL subunit is still capable of phosphorylating Mga on the EIIB^Gat^ domain cysteine to mediate CCR on the Rgg2/3 QS system. The actual phosphorylation state of the Mga PRDs remains to be demonstrated, as does how the combinatorial phosphorylation of the PRDs and EIIB^Gat^ affects the expression of Mga-dependent virulence genes.

Cysteine is one of the most nucleophilic amino acids within the proteome and its reactivity can support a variety of post-translational modifications (PTMs) such as alkylation and oxidation (disulfide bridges) through the -thiol group (60). Despite extensive studies highlighting the capability of cysteine residues for PTMs, it has been noted that phosphorylation of cysteine is a rare modification. To date, cysteine phosphorylation has been confirmed for PTS components (61, 62), as well as transcriptional regulators that harbor domains similar to these PTS components (5), and some global transcriptional regulators of *S. aureus,* such as SarA, MgrA, and CymR (58). For the *S. aureus* regulators, it was noted that the phosphorylation capability of cysteine in these transcriptional regulators is highly dependent on the oxidation state of cysteine residue; phosphorylation of cysteine is only supported when it is in the reduced state (58). This raises an interesting possibility that the highly conserved cysteine residue within the PrfA motif could potentially double as a redox sensor in addition to its role as a signal receiver for carbohydrate availability to further tailor PrfA activity. The reducing environment of host cytosol coupled with the endogenous production of GSH and the import of host GSH by *Lm* as well as the lack of CCR might all combine to fully induce PrfA activation and virulence gene expression once *Lm* reaches the cytosol (63, 64).

In summary, we have provided genetic evidence to suggest that two structurally distinct transcriptional regulators of virulence from two different Gram-positive pathogens contain a novel conserved Aro-Cys-(Aro/Xle)-Aro motif that enables them to translate the signal of carbohydrate availability to appropriately regulate the expression of virulence genes required for host infection. Although it remains to be determined empirically if the cysteine residue of these two transcriptional regulators is indeed phosphorylated (57, 58), the genetic approaches using phospho-mimetic and phospho-ablative amino acid substitutions support the notion that the cysteine within this novel Aro-Cys-(Aro/Xle)-Aro motif serves as a site of phosphorylation with regulatory consequences. Interestingly, this motif appears to be conserved in other transcriptional regulators found in both pathogenic and non-pathogenic Gram-positive species, suggesting that it may be a universally conserved mechanism to allow the bacterial cell to sense the carbohydrate availability and tailor gene expression to optimize their survival.

## SUPPLEMENTAL MATERIAL

Supplemental material spectrum.00485-24-s0001.docx Fig. S1 and S2; Tables S1 to S4.

ASM does not own the copyrights to Supplemental Material that may be linked to, or accessed through, an article. The authors have granted ASM a non-exclusive, world-wide license to publish the Supplemental Material files. Please contact the corresponding author directly for reuse.
