# Supplementary material for: Novel motif associated with carbon catabolite repression in two major Gram-positive pathogen virulence regulatory proteins
Source: Microbiol Spectr. 2024 Oct 10;12(11):e00485-24. doi: 10.1128/spectrum.00485-24 (PMC11537053; doi:10.1128/spectrum.00485-24)
Supplement: Supplemental material — Fig. S1 and S2; Tables S1 to S4. [file spectrum.00485-24-s0001.docx]

Table S1: Alignment of EIIB components and EIIB domains in known PRD-containing transcriptional regulators and glycerol kinase from Gram-positive bacterial species

| **Species** | **Protein** | **PTS Family** | **Amino acid sequence** |
| --- | --- | --- | --- |
| *B. subtilis* | EIIB_glucose_ | Glucose | LDACI**TRLR**VTVNDQKKVDK |
| GAS | EIIB_Sucrose_ | Glucose | VAH**C**A**TRLR**VMVYDEGKID |
| GAS | EIIB_Trehalose_ | Glucose | VTH**C**A**TRMR**FVLNDNNKA |
| *B. subtilis* | EIIB_Trehalose_ | Glucose | ATH**C**V**TRLR**FALIDESKVD |
| GAS | EIIB_Maltose_ | Glucose | VDA**C**M**TRLR**VTVKDPAKVG |
| *B. subtilis* | EIIB_NAG_ | Glucose | IDH**C**A**TRLR**LTVKDTALVD |
| GAS | EIIB_Fructose_ | Galactitol | VTA**CPTG**IAHTYMAEEALK |
| GAS | EIIB_Lactose_ | Galactitol | LVL**CAGG**GTSGLLANALNKA |
| *E.coli* | EIIB_Galactitol_ | Galactitol | IVA**CGGA**VATSTMAAEEIKE |
| GAS | EIIB_3-KGO_ | Galactitol | LTA**CGNGMG**SSMVIKMKVE |
| GAS | EIIB_Cellobiose_ | Galactitol | MLV**CNAG**MSTSMLVTKMQK |
| GAS | EIIB_Galactose_ | Galactitol | LAA**CGAG**VNSSHQIKDAIET |
| GAS | EIIB_Mannitol_ | Galactitol | VTV**CGNGIG**SSLLLRMKVEA |
| Lm | EIIB^Gat^ of ManR | **–** | ITI**CATGEG**TAEKLQLFIENI |
| *B. subtilis* | EIIB^Gat^ of LicR | **–** | IIV**CASGAG**SAQLLREKLRS |
| *B. subtilis* | EIIB^Gat^ of MtlR | **–** | LVV**CSSGIG**SSKMLASRLKK |
| GAS | Mga (Type-I) | **–** | LT**YAFF**ITWENSFLKVNQKD |
| GAS | Mga (Type II) | **–** | LT**YCFF**ITWENSFLKVNQKD |
| *B. subtilis* | Glycerol Kinase | **–** | VD**YHFF**GKNIPIAGAAGDQQ |
| GAS | Glycerol Kinase | **–** | AA**FHFY**GGEVPISGMAGDQQ |

Abbreviations: GAS; Group A Streptococcus. Lm; Listeria monocytogenes. NAG; N-acetylglucosamine. 3-KGO; 3-alpha ketoglutarate.

Figure S1


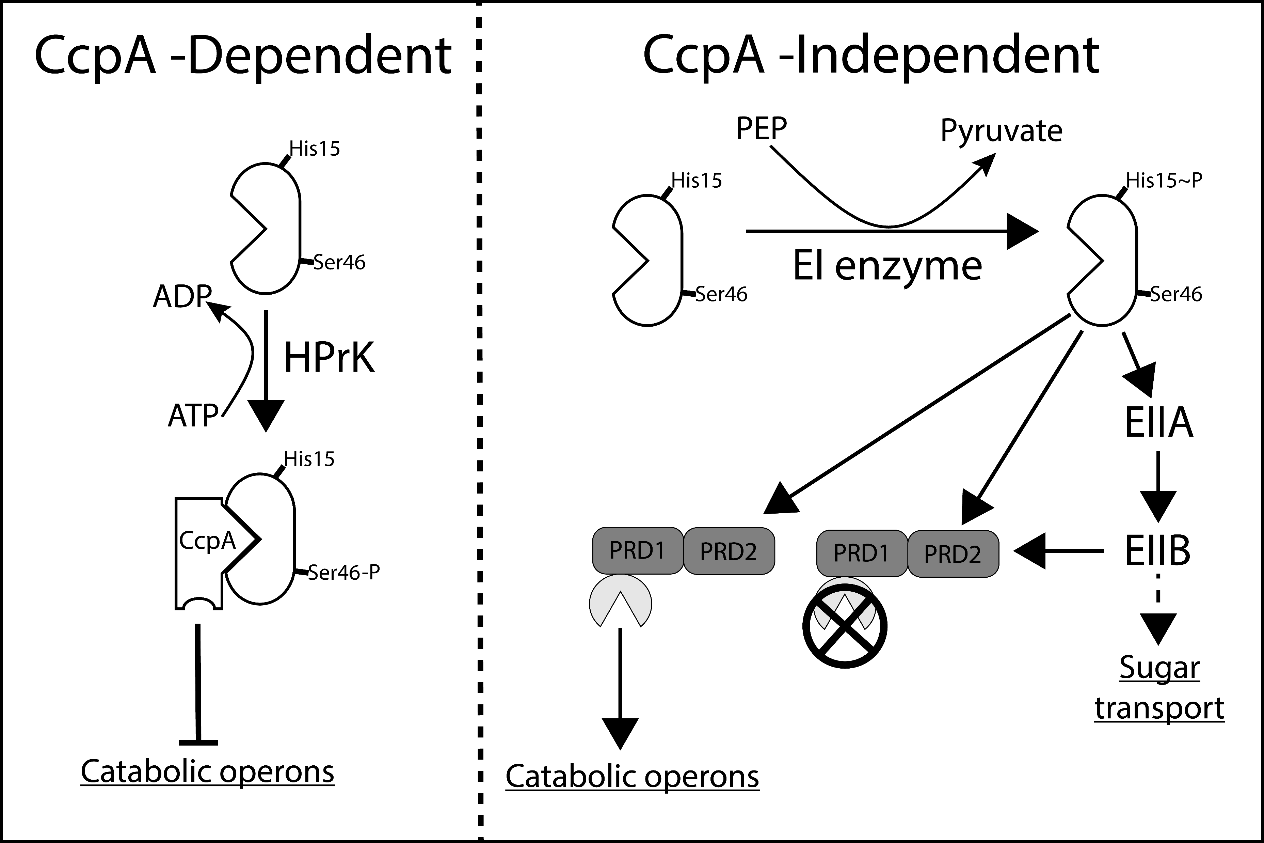


A diagram depicting how carbon catabolite repression is exerted in Gram positive bacterial species as either CcpA-dependent or CcpA-independent pathway.

Figure S2


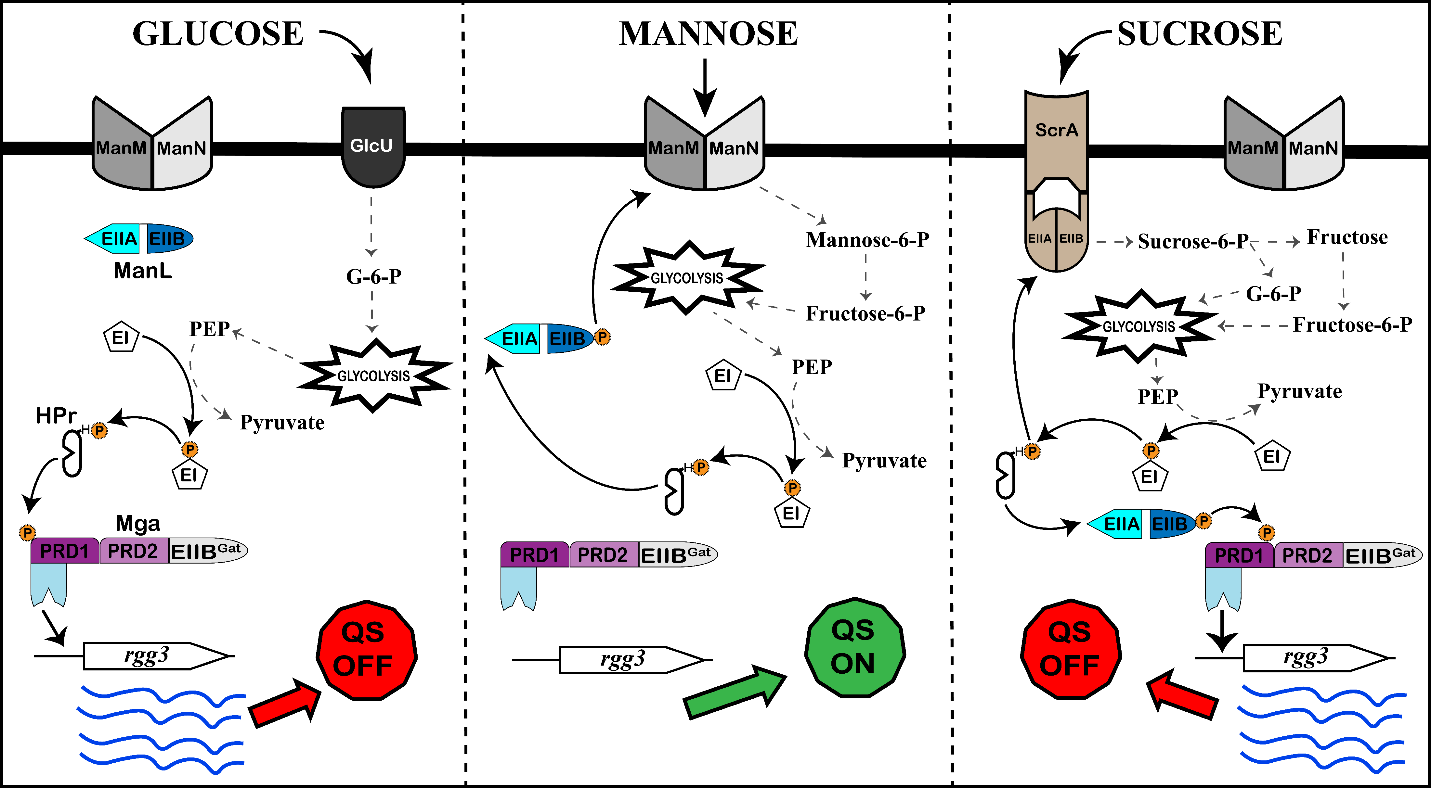


A proposed model how Rgg2/3 QS system is affected through Mga in a carbohydrate dependent manner.

Table S2: Strains list

| Strain Name | Genotype | Reference |
| --- | --- | --- |
| ***Streptococcous pyogenes* (GAS)** |  |  |
| *Streptococcus pyogenes* NZ131 (M49 Serotype) | Wild type; CovRS^+^ | (Simon & Ferretti, 1991) |
| *S. pyogenes* Δ*mga* | NZ131 Δ*mga* | (Woo *et al.*, 2022) |
| *S. pyogenes* Δ*manL* | NZ131 Δ*manL* | (Woo *et al.*, 2022) |
| *S. pyogenes* Δ*mga*::*mga*^C388S^ | NZ131 Δ*mga*::*mga* C388S | This study |
| *S. pyogenes* Δ*mga*::*mga*^C388E^ | NZ131 Δ*mga*::*mga* C388E | This study |
| *S. pyogenes* Δ*manL* Δ*mga*::*mga*^C388E^ | NZ131 Δ*manL* Δ*mga*::*mga* C388E | This study |
|  |  |  |
| ***Listeria monocytogenes* (LMO)** |  |  |
| *Listeria monocytogenes* 10403S | Wild type | (Bishop & Hinrichs, 1987) |
| *L.monocytogenes* (NF-L476) | *actA-gus-plcB* fusion | (Shetron-Rama *et al.*, 2003) |
| *L. monocytogenes* (NF-L1003) | Δ*prfA* *actA-gus-plcB* fusion | (Wong & Freitag, 2004) |
| *L. monocytogenes* (NF-L1011) | pPL2-*prfA* L140F(pNF-1019) in Δ*prfA* strain NF-L1003 | (Wong & Freitag, 2004) |
| *L. monocytogenes* (NF-L1041) | pPL2-WT *prfA* (pNF-1019) in Δ*prfA* strain NF-L1003 | (Wong & Freitag, 2004) |
| *L. monocytogenes* (DP-L1942) | Δ*actA* | (Brundage *et al.*, 1993) |
| *L. monocytogenes* (JWL0013) | pPL2-*prfA* C38E (pJW160) in Δ*prfA* strain NF-L1003 | This study |
| *L. monocytogenes* (JWL0015) | pPL2-*prfA* C38S (pJW159) in Δ*prfA* strain NF-L1003 | This study |
|  |  |  |
| *Escherichia coli* BH10C | F– *mcrA* Δ(*mrr-hsdRMS-mcrBC*) φ80*lacZ*ΔM15 Δ*lacX74 recA1 endA1 araD139* Δ (*ara-leu*)7697 *galU galK* λ– *rpsL*(Str^R^) *nupG zad::*Tn*10* *pcnB80* | (Howell-Adams & Seifert, 2000) |

Table S3: Plasmid List

| Plasmid name | Notes | Reference |
| --- | --- | --- |
| pJW107 | Erm^R^, temperature-sensitive, pWV01 *ori,* WT mga cloned in | (Woo *et al.*, 2022) |
| pNF1019 | CmR, pPL2 site specific integration vector with full length *prfA* and all promoters | (Wong & Freitag, 2004) |
| pJW146 | Complementation of *mga* with C388S | This study |
| pJW147 | Complementation of *mga* with C388E | This study |
| pJW159 | Complementation of *prfA* with C38S | This study |
| pJW160 | Complementation of *prfA* with C38E | This study |

Table S4: Primer List

| Primer name | Sequence | Notes |
| --- | --- | --- |
| JWP0007 | CGTGAGAATGTTACAGTCTATCC |  |
| JWP0008 | GAGGAAATAATTCTATGAGTCGCTTTTG |  |
| JWP0029 | GCCAGCCTAGCATGGAAAATACG |  |
| JWP0030 | CAGCTGCAAGTTCTTGTTGTTTTGCTG |  |
| JWP0083 | GATCAATCAGCTCACTTAT**AGT**TTCTTTATTACCTG | Pair with JWP0085 to mutate C388 to serine |
| JWP0084 | GATCAATCAGCTCACTTAT**GAA**TTCTTTATTACCTG | Pair with JWP0085 to mutate C388 to glutamic acid |
| JWP0085 | AGTTCTTTGCTAACTTGAGCATTATCTGC |  |
| JWLP0014 | CACAAGAATAT**TCT**ATTTTTCTATATGATGGTATC | Pair with JWLP0016 to mutate C38 to serine |
| JWLP0015 | CACAAGAATAT**GAA**ATTTTTCTATATGATGGTATC | Pair with JWLP0016 to mutate C38 to glutamic acid |
| JWLP0016 | GATCCCATTGGTTAAAAATAAGTTCTTTTTTATG |  |
| PL95 | ACATAATCAGTCCAAAGTAGATGC |  |
| NC16 | GTCAAAACATACGCTCTTATC |  |

References

Bishop, D.K., and Hinrichs, D.J. (1987) Adoptive transfer of immunity to *Listeria monocytogenes*. The influence of in vitro stimulation on lymphocyte subset requirements. *The Journal of Immunology* **139**: 2005-2009.

Brundage, R.A., Smith, G.A., Camilli, A., Theriot, J.A., and Portnoy, D.A. (1993) Expression and phosphorylation of the *Listeria monocytogenes* ActA protein in mammalian cells. *Proceedings of the National Academy of Sciences* **90**: 11890-11894.

Howell-Adams, B., and Seifert, H.S. (2000) Molecular Models Accounting For The Gene Conversion Reactions Mediating Gonococcal Pilin Antigenic Variation. *Molecular Microbiology* **37**: 1146-1158.

Shetron-Rama, L.M., Mueller, K., Bravo, J.M., Bouwer, H.G.A., Way, S.S., and Freitag, N.E. (2003) Isolation of *Listeria monocytogenes* mutants with high-level in vitro expression of host cytosol-induced gene products. *Molecular Microbiology* **48**: 1537-1551.

Simon, D., and Ferretti, J.J. (1991) Electrotransformation of *Streptococcus pyogenes* With Plasmid And Linear DNA. *FEMS Microbiology Letters* **82**: 219-224.

Wong, K.K.Y., and Freitag, N.E. (2004) A Novel Mutation within the Central *Listeria monocytogenes* Regulator PrfA That Results in Constitutive Expression of Virulence Gene Products. *Journal of Bacteriology* **186**: 6265-6276.

Woo, J.K.K., McIver, K.S., and Federle, M.J. (2022) Carbon Catabolite Repression on the Rgg2/3 Quorum Sensing System in *Streptococcus pyogenes* is Mediated by PTS^Man^ and Mga. *Molecular Microbiology* **117**: 525-538.
